# Reply to: Rethinking disease eradication: putting countries first

**DOI:** 10.1093/inthealth/ihab055

**Published:** 2021-09-16

**Authors:** Donald R Hopkins, Kashef Ijaz, Adam Weiss, Sharon L Roy, David A Ross

**Affiliations:** The Carter Center, 453 John Lewis Freedom Parkway, Atlanta, GA 30307, USA; The Carter Center, 453 John Lewis Freedom Parkway, Atlanta, GA 30307, USA; The Carter Center, 453 John Lewis Freedom Parkway, Atlanta, GA 30307, USA; Centers for Disease Control and Prevention, 1600 Clifton Road NE, Mailstop H24-3, Atlanta, GA 30329, USA; The Task Force for Global Health, 330 W. Ponce de Leon Avenue, Decatur, GA 30030, USA

**Keywords:** dracunculiasis, elimination, epidemiology, eradication, Guinea worm

## Introduction

In a recent article, Gebre^1^ suggests that endemic countries should lead in deciding on disease eradication initiatives and asserts that ‘elimination as a public health problem’ is the preferred option because eradication occurs at the expense of other health programs and weakens fragile health systems. The author's primary example is dracunculiasis (Guinea worm) eradication, but he fails to take into account that vertical disease elimination and eradication programs also strengthen health systems overall. Eradication means permanent reduction to an incidence of zero of a disease worldwide, while elimination as a public health problem means minimal disease incidence but control measures are still necessary.

## Who decides?

All countries should decide on eradication efforts since all affected countries must participate to achieve eradication. The annual World Health Assembly (WHA), which sets global targets for eradication, includes ministers of health or their representatives from all member countries. Uganda and five other dracunculiasis-endemic countries introduced the 1986 resolution in which the WHA endorsed dracunculiasis ‘elimination’ for the first time; the 1988 meeting of African ministers of health first adopted a target date of 1995. The WHA endorsed four additional resolutions on dracunculiasis, all supported by ministries of health of the endemic countries. Endemic countries further declared their support for Guinea worm eradication through the Khartoum (2002) and Geneva (2004) Declarations.

## Why is dracunculiasis eradication taking longer and costing more than smallpox eradication?

Smallpox was eradicated in 1980 after 10 y and a cost of US$300 million (almost US$1 billion in 2021, adjusted for inflation), thanks to an excellent vaccine and the disease's 2-week-long incubation period. Polio eradication, also with a 2-week-long incubation period, but a more cumbersome vaccine, is ongoing after 32 y at a cost of US$17 billion between 1988 and 2019.^2^ Dracunculiasis eradication continues after 35 y, with no vaccine or treatment and a 1-year-long incubation period at a cost so far of US$500 million.

It took 260 incubation periods to eradicate smallpox. Polio eradication has been under way for >800 incubation periods. The Guinea Worm Eradication Program (GWEP) has reduced dracunculiasis by >99% in 35 incubation periods.

## Why was there a shift away from safe water provision as a primary intervention against dracunculiasis per the original WHA resolution?

The global GWEP began by piggy-backing on the International Drinking Water Supply and Sanitation Decade (1981–1990), but that decade did not achieve its goal to provide clean drinking water to all (which would have eradicated dracunculiasis and provided other benefits) despite multibillion dollar backing from the United Nations and many international donors.^3^ The dracunculiasis campaign continued after the decade ended by helping countries use less-expensive interventions to stop transmission of a disease that affected people's agricultural productivity, school attendance and health. The GWEP also advocates for prioritizing Guinea worm–endemic villages for borehole wells and to repair existing water systems.

## Why not invest in broader health services instead?

Continued improvements in standards of living will improve health systems, but such changes take decades and should not hinder faster improvements by disease eradication. Many health systems are fragile because politically powerless populations are denied their fair share of resources. As long as the neglect persists, neglected populations will suffer, whether resources for broader health services are available or not.

Basic health services alone usually are not sufficient to control or eliminate neglected tropical diseases, including dracunculiasis. Eradication programs encourage governments to provide some services everywhere the problem exists. For example, in South Sudan, the government built up the rural public health system on the skeleton of the GWEP.

## Why not pursue elimination as a public health problem instead of disease eradication?

Eliminating a disease as a public health problem leaves a risk of resurgence. Eradication does not. Ghana reduced yaws to low levels, but routine health services let yaws resurge.^4^ Eradication ends the risk and suffering of a disease forever, while avoiding the costs required to maintain control indefinitely. After several years of controlling onchocerciasis (river blindness) with annual mass drug administration, Uganda opted instead to eliminate onchocerciasis transmission and is now close to achieving it.^5^

## The way forward?

It is a false choice to require deciding between focusing on a single disease and broader health programs. It is possible to do both while helping neglected people achieve better health. With political resolve we can assist neglected populations, aim for improved basic health services and apply the data-driven, outcome-oriented discipline of single-disease programs. Single-disease programs provide benefits beyond their main focus, in trained workers, surveillance and response infrastructure and logistics, as, for example, some single-disease programs are doing now to help combat coronavirus disease 2019. With a similar focus on outcomes, quantitative targets and commitment to equity, broader health services could be as effective and attractive as single-disease programs.

## Data Availability

None.
